# Involvement of Transcription Factor NR2F2 in Human Trophoblast Differentiation

**DOI:** 10.1371/journal.pone.0009417

**Published:** 2010-02-25

**Authors:** Michael A. Hubert, Susan L. Sherritt, Cindy J. Bachurski, Stuart Handwerger

**Affiliations:** 1 Department of Pediatrics, University of Cincinnati, Cincinnati, Ohio, United States of America; 2 Division of Endocrinology, Children's Hospital Medical Center, Cincinnati, Ohio, United States of America; 3 Division of Pulmonary Biology, Children's Hospital Medical Center, Cincinnati, Ohio, United States of America; CNRS, France

## Abstract

**Background:**

During the *in vitro* differentiation of human villous cytotrophoblast (CTB) cells to a syncytiotrophoblast (STB) phenotype, mRNA levels for the nuclear hormone receptor NR2F2 (ARP-1, COUP-TFII) increase rapidly, reaching a peak at day 1 of differentiation that is 8.8-fold greater than that in undifferentiated CTB cells. To examine whether NR2F2 is involved in the regulation of villous CTB cell differentiation, studies were performed to determine whether NR2F2 regulates the expression of TFAP2A (AP-2α), a transcription factor that is critical for the terminal differentiation of these cells to a STB phenotype.

**Methodology/Primary Findings:**

Overexpression of NR2F2 in primary cultures of human CTB cells and JEG-3 human choriocarcinoma cells induced dose-dependent increases in TFAP2A promoter activity. Conversely, siRNA mediated silencing of the NR2F2 gene in villous CTB undergoing spontaneous differentiation blocked the induction of the mRNAs for TFAP2A and several STB cell specific marker genes, including human placental lactogen (hPL), pregnancy specific glycoprotein 1 (PSG1) and corticotropin releasing hormone (CRH) by 51–59%. The induction of TFAP2A promoter activity by NR2F2 was potentiated by the nuclear hormone receptors retinoic acid receptor alpha (RARA) and retinoid X receptor alpha (RXRA).

**Conclusions/Significance:**

Taken together, these results strongly suggest that NR2F2 is involved in villous CTB cell differentiation and that NR2F2 acts, at least in part, by directly activating TFAP2A gene expression and by potentiating the transactivation of TFAP2A by RARA and RXRA.

## Introduction

During human placental development, cytotrophoblast (CTB) cells differentiate into syncytiotrophoblast (STB) cells that form the outermost cell layer of the placental villus. These cells are important in many of the cellular processes that are critical for pregnancy maintenance and fetal survival, including ion, substrate, and gas transport, and hormone production. Many factors have been implicated in the regulation of villous CTB differentiation, including EGF [1], hCG [2], LIF [3], CSF-1 [4], IGF-I [5], leptin [6], cAMP [7], members of the TGFβ superfamily (including TGFβ and TGIF) [8], the Wnt/β-catenin pathway [9], [10], and the transcription factors PPARγ [11], Ikaros [12], GATA-2/3 [13], RARA [14] and RXRA [15]. However, relatively little is known about the cellular mechanisms by which these factors regulate CTB differentiation.

Several lines of evidence suggest that the transcription factor NR2F2 ((nuclear receptor subfamily 2, group F, member 2, also known as ARP-1 (apolipoprotein repressor protein 1) and COUP-TFII (chicken ovalbumin upstream protein TFII)), a member of the nuclear hormone receptor gene family, may also be involved in the regulation of villous CTB differentiation. NR2F2 is expressed in many tissues, including skin, kidney, lung, stomach, intestine, salivary gland, pancreas, testes, ovary, uterus, prostate and placenta [16]. NR2F2 has been shown to have many actions in reproductive tissues. For example, NR2F2 in the uterus is a downstream target of the Indian Hedgehog signaling pathway that mediates communication between uterine epithelial and stromal compartments [17]. In addition, NR2F2 in the uterus may play a role in the preparation of the uterus for implantation. Mutant females show enhanced trophoblast giant cell differentiation, reduction of the spongiotrophoblast layer, and absence of labyrinth formation due to improper vascularization of the placenta.

Studies from our laboratory strongly suggest that the retinoic acid-inducible transcription factor TFAP2A (also known as activator protein 2α or AP-2α) is also involved in the regulation of human villous CTB differentiation. We observed that TFAP2A induces the expression of the STB-specific proteins human placental lactogen (hPL) [18], human chorionic gonadotropin alpha (hCGα) [19], hCGβ [19] and corticotropin releasing hormone (CRH) [20]; and studies by others demonstrated that TFAP2A stimulates the expression of additional genes expressed in STB cells, including aromatase cytochrome P-450 (CYP11A1) [21], germ cell alkaline phosphatase [22], 17ß-hydoxysteroid dehydrogenase type 1 [23] and leucine aminopeptidase/oxytocinase [24]. In addition, we noted that 18 of the 25 most induced genes and 17 of the 20 most repressed genes during villous CTB differentiation are TFAP2A-dependent [25]. Moreover, we observed that silencing of TFAP2A expression in differentiating cytotrophoblast cells by overexpression of a dominant/negative TFAP2A protein significantly inhibits the induction of 91 of the 205 genes normally induced during villous CTB differentiation (44.4%) and blocked the repression of 34 of the 229 genes (14.9%) down-regulated during the differentiation process [26].

Since TFAP2A expression is induced by retinoic acid [27], we hypothesized that NR2F2 may regulate CTB differentiation by modulating the induction of TFAP2A by retinoic acid. To test this hypothesis, we have examined whether NR2F2 regulates the TFAP2A promoter in human villous CTB cells undergoing differentiation to a STB phenotype and whether silencing of NR2F2 expression by NR2F2 siRNAs attenuates syncytialization and the expression of STB-specific genes. In addition, we have examined the effects of NR2F2 on RARA- and RXRA-induced transactivation of the TFAP2A promoter.

## Results

**Figure 1:**
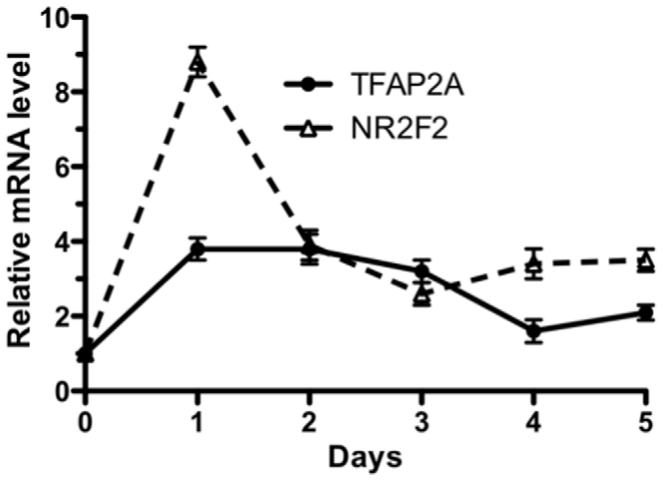
Time course of TFAP2A and NR2F2 mRNA levels during villous CTB differentiation. An enriched fraction of enzymatically dispersed villous CTB cells were cultured *in vitro* for five days as described in Methods. TFAP2A and NR2F2 mRNA levels were determined by real-time PCR at the end of each day. The amounts of TFAP2A and NR2F2 mRNAs in each culture well were normalized to the amount of GAPDH mRNA in the same sample. Each point represents the mean ± SEM of triplicate observations from 3 different placenta cell preparations (n = 3 wells/placenta culture).

To determine whether NR2F2 mRNA is expressed in the early stages of villous CTB differentiation, NR2F2 mRNA levels were measured at daily intervals during 5 days of spontaneous differentiation; and the pattern of NR2F2 mRNA was compared to that of TFAP2A mRNA. As shown in Figure 1, NR2F2 mRNA levels increased markedly during the differentiation process, reaching a peak at 1 day that was 8.8-fold greater than baseline levels. The levels for NR2F2 mRNA then decreased by 50% over the next day, plateauing at levels that were about 4-fold greater than baseline. TFAP2A mRNA levels increased 3.8-fold by day 1, plateaued until day 3 and then decreased to levels that were about 2-fold greater than baseline.

**Figure 2:**
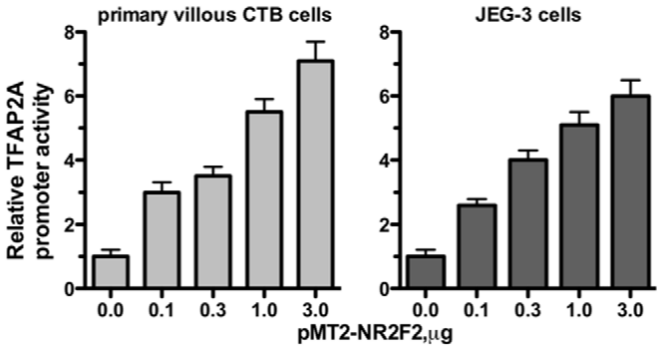
The effect of NR2F2 on TFAP2A promoter activity. Primary villous CTB cells (left) and JEG-3 cells (right) were co-transfected with pGL3β-TFAP2A-Luc and pMT2-NR2F2 as described in Methods. The amount of luciferase activity in each sample was normalized to the amount of Renilla luciferase activity. Each bar represents the mean of triplicate observations; and the bars enclose 1 SEM. Stimulation of TFAP2 promoter activity by pMT2-NR2F2 was observed in three other experiments using primary CTB cells and JEG-3 cells.

To examine whether NR2F2 transactivates the TFAP2A promoter, primary cultures of an enriched fraction of human cytotrophoblast cells and JEG-3 human choriocarcinoma cells were co-transfected with an expression plasmid for NR2F2 (pMT2-NR2F2) and a plasmid containing the TFAP2A promoter coupled to a luciferase reporter gene (pGL3β-TFAP2A-Luc). Control cells were co-transfected with an “empty” expression plasmid (pMT2) and pGL3β-TFAP2A-Luc. As shown in Figure 2, pMT2-NR2F2 stimulated dose-dependent increases in TFAP2A promoter activity in both the primary CTB cells and JEG-3 cells. The luciferase activities of the CTB cells and JEG-3 cells co-transfected with 3.0 µg pMT2-NR2F2 were 7.1±0.6 and 6.0±0.5-fold greater respectively than that of control cells (P<0.001 in each instance).

**Figure 3:**
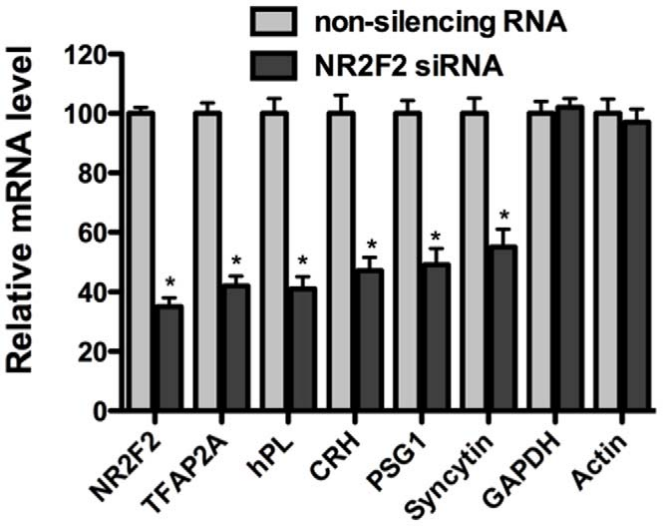
The effects of NR2F2 gene silencing on TFAP2A mRNA levels and the mRNA levels of the syncytiotrophoblast cell markers, hPL, CRH, PSG1 and syncytin. Human cytotrophoblast cells were transfected in two separate experiments with a NR2F2 siRNA or a non-silencing (control) RNA as described in Methods. The cultures were terminated 48 h later, total RNA was extracted, and quantitative real-time PCR was performed for the indicated genes. In each instance, the amount of mRNA for each gene was normalized to the amount of GAPDH mRNA in the same sample. The final results for each gene from the two experiments (n = 6) are expressed as the % change of the normalized mRNA in the NR2F2 siRNA-exposed cells relative to the silent (control) RNA ((silent RNA- siRNA)/silent RNA)x100)). The bars represent the % change in mRNA levels from the two experiments; the error bars represent ± 1 SEM (n = 6). Each of the mRNAs in the NR2F2 siRNA-exposed cells, with the exception of GAPDH mRNA, was significantly less than that in the silent RNA-exposed cells (P<0.001 to <0.005 in each instance). Similar results were observed in 2 other independent experiments. *<0.01

Conversely, silencing of NR2F2 expression with a NR2F2 siRNA attenuated the induction of TFAP2A expression during CTB cell differentiation as well as the expression of other genes that are specific markers of STB differentiation. As shown in Figure 3, silencing of the NR2F2 gene in villous cytotrophoblast cells undergoing differentiation significantly attenuated expression of the TFAP2A gene and the genes for hPL, pregnancy specific glycoprotein 1 (PSG1) and corticotropin releasing hormone (CRH), all of which are markedly induced during the differentiation process. In two experiments, exposure of CTB cells for 16 h repressed NR2F2 mRNA levels by 65.3±3.0% (n = 6) and repressed TFAP2A mRNA levels by 58.2±3.4% (p<0.001 in each instance) as compared to cells exposed to the non-silencing RNA control. The mRNA levels for hPL, CRH, and PSG1 in the two experiments were repressed by 51.3 to 59.4% (p<0.001 in each instance), while GAPDH and actin mRNA levels were unaffected. The mRNA levels of syncytin, a transmembrane glycoprotein critical for the fusion of CTB cells [28], [29], were repressed by 45.0±5.0% (p<0.001).

**Figure 4:**
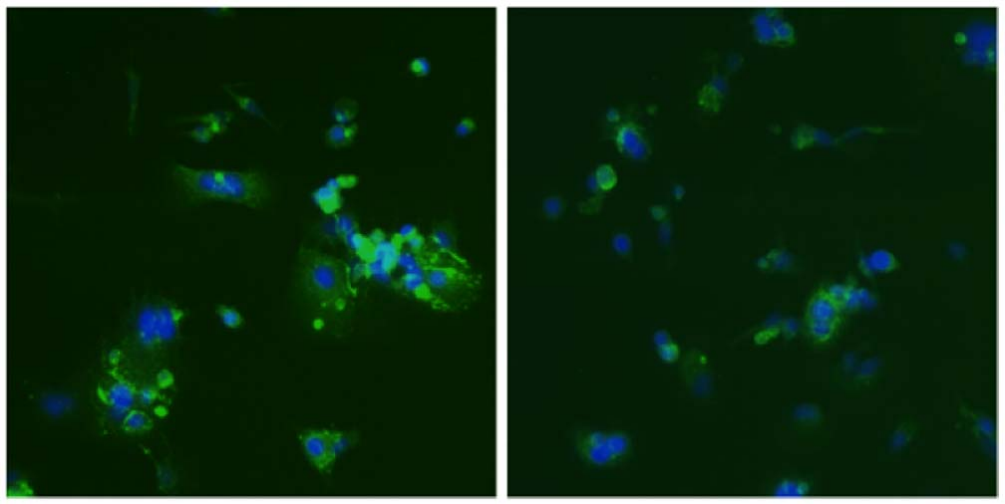
The effect of NR2F2 gene silencing on syncytialization of villous CTB cells. Human cytotrophoblast cells were transfected with a non-silencing (control) RNA (left panel) or a NR2F2 siRNA (right panel) as described in Figure 3. The cultures were terminated 4 days later and the cell membranes were stained with an anti-desmosomal protein antiserum (green), and the nuclei were stained with DAPI (blue) as described in Methods. 10× magnification

Silencing of NR2F2 expression with a NR2F2 siRNA also attenuated the syncytialization of CTB cells. After 4 days of exposure to the non-silencing RNA control, 58% of DAPI-positive nuclei (1000 counted) were in multinucleated cells, and most of the multinucleated cells contained 3 or more nuclei. In contrast, only 18% of the DAPI-positive nuclei of the CTB exposed to the NR2F2 siRNA were multinucleated; and most of these multinucleated cells contained only 2 or 3 nuclei. Representative microscopic fields (1ox) of CTB cells exposed to the non-silencing RNA control (left) and NR2F2 siRNA (right) are shown in Figure 4.

**Figure 5:**
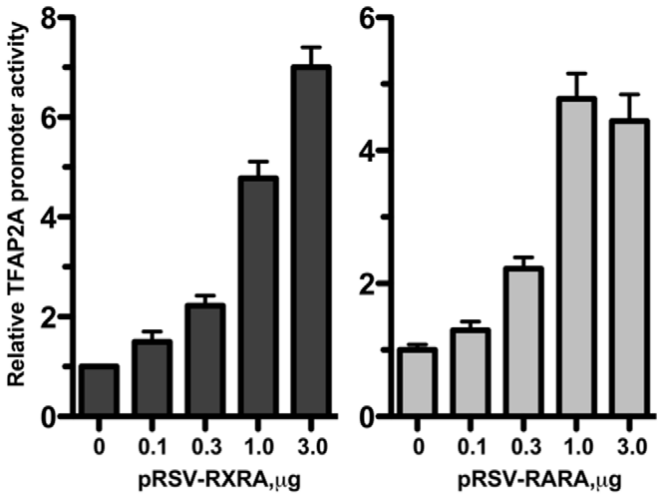
The effect of RXRA and RARA on TFAP2A promoter activity. JEG-3 cells were co-transfected with pGL3β-TFAP2A-Luc and pRSV-RXRA or pRSV-RARA at the amounts indicated in the Figure. Each bar represents the mean of triplicate observations; and the bars enclose 1 SEM. Similar results were observed in two additional experiments.

To identify a potential mechanism for how NR2F2 activates the TFAP2A promoter, the ability of NR2F2 to potentiate the RXRA and RARA mediated regulation of TFAP2A promoter activity was tested in transient transfection of JEG3 cells that have minimal expression of all 4 genes at baseline. As shown in Figure 5, pRSV-RXRA (left panel) and pRSV-RARA (right panel) induced dose-dependent increases in TFAP2A promoter activity in JEG3 cells. NR2F2 alone induced TFAP2A promoter activity by approximately 4 fold in JEG3 cells. JEG3 cells co-transfected with pGL3β-TFAP2A-Luc and pRSV-RARA (3.0 µg) expressed 4.5±0.4 (mean ± SEM) fold greater luciferase activity than cells co-transfected with pGL3β-TFAP2A-Luc and pRSV. JEG3 cells co-transfected with pGL3β-TFAP2A-Luc and pRSV-RXRA (3.0 µg) expressed 7.0±0.4 fold greater luciferase activity than cells co-transfected with pGL3β-TFAP2A-Luc and pRSV in the absence of pMT2-RXRA.

**Figure 6:**
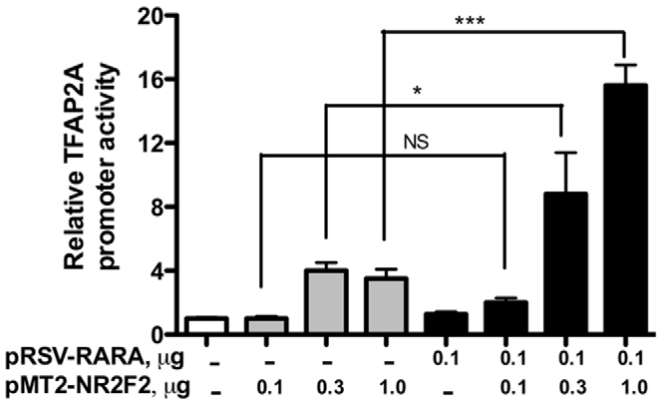
The effect of RARA on NR2F2-induced TFAP2A promoter activity. JEG-3 cells were co-transfected with pGL3β-TFAP2A-Luc and pMT2-NR2F2 in the presence and absence of pRSV-RARA. An equivalent amount of the empty pRSV plasmid was co-transfected into the cells that were not co-transfected with pRSV-RARA. Each bar represents the mean of triplicate wells; and the brackets enclose 1 SEM. NS = not significant; * = p<0.05; *** = p<0.001. Similar results were obtained in two other experiments.

**Figure 7:**
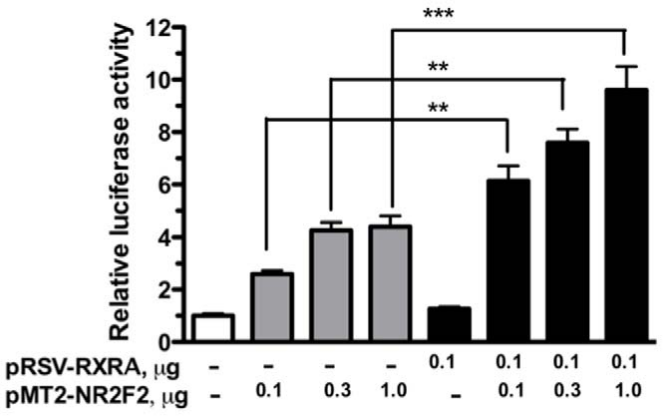
The effect of RXRA on NR2F2-induced TFAP2A promoter activity. JEG-3 cells were co-transfected with pGL3β-TFAP2A-Luc and pMT2-NR2F2 in the presence and absence of pRSV-RXRA. An equivalent amount of the empty pRSV plasmid was co-transfected into the cells that were not co-transfected with pRSV-RXRA. Each bar represents the mean of triplicate wells; and the brackets enclose 1 SEM. NS = not significant; * = p<0.05; *** = p<0.001. Similar results were obtained in two other experiments.

Since NR2F2 has been shown to attenuate or potentiate the transactivation of several genes by nuclear hormone receptors, experiments were next performed to determine whether NR2F2 modulates the transactivation of the TFAP2A promoter by RARA and RXRA. In the experiment depicted in Figure 6, JEG3 cells were co-transfected with increasing amounts of pMT2-NR2F2 in the absence and presence of a constant amount of pRSV-RARA (0.1 µg) that has little effect itself on TFAP2A promoter activity. The JEG3 cells co-transfected with pRSV-RARA (0.1 µg) and pMT2-NR2F2 at doses of 0.1, 0.3 and 1.0 µg expressed 2.0, 2.4 and 4.5-fold greater luciferase activity, respectively, than the cells exposed to pMT2-NR2F2 alone. In the experiment depicted in Figure 7, JEG3 cells were co-transfected with increasing amounts of pMT2-NR2F2 in the absence and presence of a constant amount of pRSV-RXRA (0.1 µg) that has little effect itself on TFAP2A promoter activity. The JEG3 cells co-transfected with pRSV-RXRA (0.1 µg) and pMT2-NR2F2 at doses of 0.1, 0.3 and 1.0 µg expressed 2.4, 1.8 and 2.2-fold greater luciferase activity, respectively than the cells exposed to pMT2-NR2F2 alone.

## Discussion

**Figure 8:**
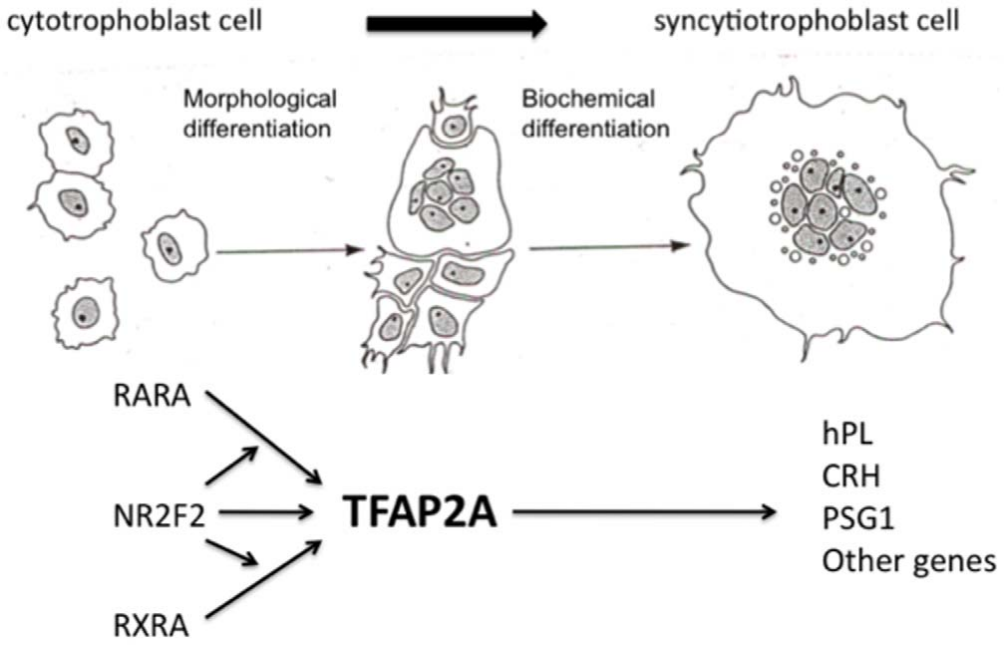
Schematic representation for the role of NR2F2 in the differentiation of human villous CTB cells to a STB cell phenotype. NR2F2 transactivates the TFAP2A promoter and potentiates the transactivation of the promoter by RARA and RXRA. Activation of TFAP2A in turn modulates the expression of downstream genes involved in terminal STB cell differentiation.

This study demonstrates that the transcription factor NR2F2 is involved in the *in vitro* differentiation of human CTB cells to a STB cell phenotype. NR2F2 transactivated the TFAP2A promoter in human CTB cells and JEG-3 choriocarcinoma cells and potentiated the transactivation of the TFAP2A promoter by RARA and RXRA. Furthermore, silencing of the NR2F2 gene in cultured CTB cells undergoing spontaneous differentiation markedly inhibited the expression of TFAP2A mRNA and the expression of the STB-specific marker genes hPL, CRH and PSG1. Silencing of NR2F2 also inhibited the syncytialization of CTB cells, as shown by immunocytochemistry using an antibody to desmosomal protein, and the expression of syncytin mRNA. Earlier studies demonstrated that the transmembrane glycoprotein syncytin, which is derived from the envelope protein of the human retrovirus HERV-W, is critical for the syncytialization of CTB cells [28], [29]. Inhibition of syncytin expression has been shown to block CTB fusion and overexpression of syncytin has been shown to cause the fusion of cells that do not normally fuse. Taken together, these observations strongly suggest a critical role for NR2F2 in the regulation of CTB differentiation, both by direct transactivation of the TFAP2A promoter and by potentiating the transactivation of the TFAP2A promoter in response to RARA and RXRA. A schematic representation of the role for NR2F2 in villous CTB differentiation is shown in Figure 8.

In an earlier report, we noted that NR2F2 attenuated both the basal activity of the hPL promoter and the transactivation of the promoter in response to thyroid hormone receptor beta (THRB) and RARA [30]. These experiments, performed in 1996, used a CAT (chloamphenicol acetytransferase) reporter plasmid ((pChlorAceE (US Biochemical, Cleveland, OH)) containing a 2.3 kb (nt −2300 to +2) fragment of the hPL (hCS-A) promoter. However, when we recently repeated these experiments using a pGL3β-Luciferase reporter plasmid (Promega) containing the same hPL promoter fragment we noted that NR2F2 induced the basal activity of the promoter and potentiated rather than attenuated hPL promoter activity in response to THRB and RARA (data not shown). Although the reasons for the differences between these studies are unclear, the observation that silencing of the NR2F2 gene represses hPL mRNA levels in human trophoblast cells strongly suggests that NR2F2 induces the hPL promoter. The findings observed with the old CAT reporter vectors most likely result from artifactual influences from pBR322 vector derived sequences [31], [32]. Many of these sequences have been removed from the newer luciferase vectors. The pGL3β vector also has an upstream poly A signal to block aberrant vector initiated transcription. Since all transient transfection experiments have the potential to give spurious results, we used siRNA knockdown of endogenous NR2F2 expression to corroborate our current findings.

Most members of the nuclear hormone receptor family have a high degree of homology in their DNA binding domain. This feature is reflected by similarities in the nucleotide sequences of the steroid response elements (SREs). The SRE consists of two copies of the half-site AG(G/T)T(C/G)A, with the number, spacing, and orientation of these motifs in many instances determining the specificity and strength of the response element. Specificity of the SREs in many genes is determined by the base-pair spacing between the half-site repeats, i.e. a spacing of 1, 3, 4 or 5 nucleotides (DR-1, 3, 4 or 5) has been reported to specify a retinoid X responsive element (RXRE), vitamin D response element (VDRE), thyroid hormone response element (TRE) or a retinoic acid responsive element (RARE), respectively [33]. RXRs have been reported to form heterodimers with RARs, TRs and VDR and enhance the binding affinities of these nuclear hormone receptors to DNA SREs [34], [35]. Like RXR, NR2F2 binds preferentially to an AGGTCA-like DR-1 site on many NR2F2 responsive genes [35], [36].

While it has been known for many years that TFAP2A is induced by retinoic acid, the molecular mechanism by which retinoic acid induces transcription of the TFAP2A gene is poorly understood. Analysis of the TFAP2A DNA sequence indicates that there are no consensus nuclear hormone receptor binding sites. Nucleotides −5 to −2 of the proximal promoter have the sequence ACTT, but site directed mutagenesis of this site had no significant effect on transactivation (data not shown). Activation of transcription by NR2F2, however, in some instances may not require DNA binding. Malik and Karathanasis [37] and Power and Cereghini [38] suggested that NR2F2-mediated activation results from direct interactions of the NR2F2 activation domains with components of the basal transcription machinery, specifically TF-IIB. Wang, Bai and co-workers [39] demonstrated that NR2F2 inhibits hTERT transcription by binding to the region of the hTERT promoter at bases nt −201 to +35 that contains an E-box motif (CACGTG). The suppression of hTERT promoter activity could be reversed by c-Myc, which competed with NR2F2 for binding to the E-box. Taken together, this finding suggests that the transactivation of the TFAP2A promoter by NR2F2, RXRA and RARA could be due to binding to a different site than that previously described for steroid hormone binding or to an interaction of NR2F2 with components of the basal transcription machinery.

Early studies of NR2F2 action demonstrated that this transcription factor attenuated transactivation by RARA, RXRA and other nuclear hormone receptor family members. The inhibition of transcription by NR2F2 was mediated by two mechanisms, active repression and trans-repression. In active repression, NR2F2 binds to its response element, while in trans-repression NR2F2 inhibits transcription in the absence of its cognate binding motif or independent of nucleic acid binding [40]. Several mutants of NR2F2 that bind strongly to DNA fail to repress transcription [40]. The mechanism for the observed inhibitory property of NR2F2 is thought to be via competition with these receptors for their binding sites and by heterodimerization with RXR. It is now known that NR2F2 can also activate transcription [41], [42]. In fact, NR2F2 may have diverse actions within the same cell. For example, NR2F2 has been shown to determine hepatoma phenotype by acting both as a transcriptional repressor of microsomal triglyceride transfer protein (MTP) and an inducer of CYP7A1 [43]. Binding of NR2F2 to a conserved DR1 site of the MTP promoter represses MTP gene expression. Within the same cellular context, NR2F2 binds to the rat CYP7A1 promoter causing enhanced transcription [41]. We now show that NR2F2 potentiates RARA and RXRA mediated transcription of TFAP2A to promote the program of syncytiotrophoblast differentiation.

In summary, the findings of this study strongly suggest that NR2F2 is an important transcription factor in the induction of terminal differentiation of villous CTB cells to a STB cell phenotype. The action of NR2F2 is mediated, at least in part, by transactivating the TFAP2A promoter and by potentiating the transactivation of the TFAP2A promoter by RARA and RXRA. Since deletion of NR2F2 in mouse uterine stromal and smooth muscle cells also results in changes in placental differentiation [44], the overall effects of NR2F2 on placental development may be mediated by both its direct actions on the placenta and its effects on uterine factors that influence placental development.

## Materials and Methods

### Plasmids

TFAP2A promoter studies were performed with a human TFAP2A promoter fragment (nt −1728 to +286) linked to a luciferase reporter gene in pGL3β (Promega Corp., Madison, WI) (pGL3β-TFAP2A-Luc) [19]. An expression vector for NR2F2 (pMT2-NR2F2) was a gift from Dr. S. Karathanasis (Lederle Laboratories). Expression plasmids for RXRA (pRSV-RXRA) and RARA (pRSV-RARA) were gifts from Dr. R. Evans (Scripps Institute). The control renilla luciferase expression plasmid (pRL-TKLuc) was purchased from Promega Corp.

### Cell Culture and Transient Transfections

Highly enriched fractions of human CTB cells were prepared by enzymatic digestion of third trimester placentas, followed by purification with immunomagnetic beads coupled to an antiserum to human CD9 [20]. The protocol to collect the placentas was approved by the Institutional Review Boards of the Cincinnati Children's Hospital Medical Center and the University of Cincinnati. The cells were cultured in DMEM with 10% FBS containing penicillin, streptomycin, and amphotericin B for 3 days, at which time >95% of the mononuclear cytotrophoblast cells had aggregated and fused to form a multinucleated syncytium. JEG-3 cells were cultured in MEM (Eagle) with 2 mM L-glutamine and Earle's BSS adjusted to contain 1.5 g/L sodium bicarbonate, 0.1 mM non-essential amino acids, and 1.0 mM sodium pyruvate, 90%; fetal bovine serum, 10%. JEG-3 cells express relatively low amounts of TFAP2A.

Transient transfections were performed in triplicate in 12 well plates by the liposome method [20]. The cells (4×10^6^ cells/well) were harvested 48 hours after transfection in 1x reporter lysis buffer (Promega). Luciferase activity in each well was normalized to co-transfected renilla luciferase activity. The results are presented as the mean ± SEM of the normalized luciferase activity. The RARA and RXRA over-expression experiments were performed in the presence of all-*trans* retinoic acid (10 µM) or 9-cis retinoic acid (1 µM), respectively.

### Gene Silencing

Freshly prepared human cytotrophoblast cells (4×10^6^ cells/well) were plated in 6 well culture plates in 2 ml of keratinocyte growth media (Invitrogen, Carlesbad, CA) containing 10% FBS. Approximately 16 h later, the medium in each well was removed; and the cells were washed and then transfected with 150 nM NR2F2 siRNA or a scrambled RNA control using the commercial transfection reagent Darmafect 4 (Dharmacon, Lafayette, CO) in 2 ml of OptiMEM medium (Invitrogen). The sequence of the NR2F2 siRNA was sense r(GTG GAA TTT ATT GGC AGC CAA) and antisense r(UUG GCU GCC AAU AAA UUC C)dAdC (Qiagen, Valencia, CA). The non-silencing RNA control (sequence not provided by manufacturer) was purchased from Qiagen. At 48 h after exposure to the siRNA or non-silencing RNA, total cell RNA was isolated from the cells and analyzed by real-time PCR as described below. In each instance, the amount of mRNA for each gene was normalized to the amount of GAPDH mRNA in the same sample. The final results for each gene are expressed as the % change of the normalized mRNA in the NR2F2 siRNA-exposed cells relative to the silent (control) RNA ((silent RNA- siRNA)/silent RNA)×100)).

### RNA Analysis

Total RNA was extracted from the cells using Trizol (Invitrogen, Carlsbad, CA, USA) according to the manufacturer's specifications. Two micrograms of total RNA were reverse transcribed using SuperScript II Reverse Transcriptase (Invitrogen). Real-time PCR reactions were performed using a Stratagene Mx3000 P instrument (Stratagene, La Jolla, CA, USA). Quantitative PCR amplifications were performed using the Eppendorf HotMasterMix (Brinkmann Instruments, Westbury, NY, USA) supplemented with SYBR Green (Molecular Probes, Eugene, OR, USA) and ROX (Stratagene). In each instance, primer pairs were selected that amplified across intron-exon boundaries. The mix was used according to the manufacturer's instructions using a 20 µl final volume. PCR reactions were performed after a two minute incubation at 95°C, followed by 40 cycles at 95°C for 30 seconds, 55°C for 1 minute, and 72°C for 30 seconds. Dissociation/association curves for each reaction were determined after the 40^th^ cycle. A single dissociation curve was noted for each primer set. Preliminary experiments determined that the conditions used for each primer were optimized for these conditions. The primers used for quantitative PCR are shown in Table 1.

**Table 1:** Sequence of primers used in QPCR analyses.

Gene/accession number	Sense/antisense	Sequence
**hPL**	S	gct atg ctc caa gcc cat c
J00118	AS	tgc agg aat gaa tac ttc tgg tc
**NR2F2**	S	gcc ata gtc ctg ttc acc t
M64497	AS	gca cac tga gac ttt tcc tg
**PSG1**	S	cat ccg cag tga ccc agt
NM_006905	AS	tct cct gaa cgg taa tag gtg aa
**TFAP2A**	S	ctc aac cga caa cat tcc
NM_003220	AS	cgg tga act ctt tgc ata tc
**CRH**	S	tcc cat ctc cct gga tct c
NM_000756	AS	agc ttg ctg tgc taa ctg ctc
**GAPDH**	S	gaa ggt gaa ggt cgg agt
M33197	AS	gat ggc aac aat atc cac tt
**Beta-actin**	A	ctg gac ttc gag caa gag at
	AS	gat gtc cac gtc aca ctt ca

### Immunocytochemistry

Freshly prepared CTB cells were plated on glass cover slips in 6-well plates and cultured for 3 days with the NR2F2 siRNA or the non-silencing RNA (triplicate wells for each treatment) as described above. The cells were fixed with 80% acetone/20% PBS for 5 min and washed for 5 min with PBS containing 1% saponin (PBS-S). Following washing, the cells were blocked with 5% goat serum in PBS-S for 30 min at room temperature. A monoclonal anti-desmosomal protein antibody (Sigma, St Louis, MO) was added to the blocking solution at a 1∶400 dilution and the cells were incubated at 37°C for 2 h. Controls were performed with normal rabbit serum at a 1∶400 dilution or by leaving out the primary antibody. Subsequently, the cells were washed with PBS-S and incubated with FITC-goat antimouse IgG (Invitrogen, Carlsbad, CA) at 37°C in the dark for 3.5 h. Three additional washes were performed with PBS for 5 min each. Cell nuclei were visualized by counterstaining with 0.1 mg/ml DAPI (40,6-diamidine-20-phenylindole, dihydrochloride) (Molecular Probes, Eugene, OR) for 5 min at room temperature. The cover slips were inverted and mounted with glycerol on microscope slides and photographed at a magnification of 10×. The percentage of nuclei in multinucleated vs mononuclear cells in the 2 treatment groups was determined by examining 1000 nuclei in each group.

### Statistical Analysis

Multiple comparisons were performed by one way ANOVA or repeated measures ANOVA together with post-hoc pairwise comparisons. The values were expressed as the mean ± SEM, and *P*<0.05 was considered statistically significant.
